# Muscle Oxygenation, Heart Rate, and Blood Lactate Concentration During Submaximal and Maximal Interval Swimming

**DOI:** 10.3389/fspor.2021.759925

**Published:** 2021-12-13

**Authors:** Athanasios A. Dalamitros, Eleni Semaltianou, Argyris G. Toubekis, Athanasios Kabasakalis

**Affiliations:** ^1^Laboratory of Evaluation of Human Biological Performance, School of Physical Education and Sport Sciences, Aristotle University of Thessaloniki, Thessaloniki, Greece; ^2^School of Physical Education and Sport Science, National and Kapodistrian University of Athens, Athens, Greece

**Keywords:** physiological testing procedures, near-infrared spectroscopy (NIRS), interrelationship, different intensity protocols, interval swimming

## Abstract

This study aimed to determine the relationship between three testing procedures during different intensity interval efforts in swimming. Twelve national-level swimmers of both genders executed, on different occasions and after a standardized warm-up, a swimming protocol consisting of either a submaximal (Submax: 8 efforts of 50 m) or a maximal interval (Max: 4 efforts of 15 m), followed by two series of four maximal 25 m efforts. Near-infrared spectroscopy in terms of muscle oxygen saturation (SmO_2_), heart rate (HR), and blood lactate concentration (BLa) were analyzed at three testing points: after the Submax or the Max protocol (TP_1_), after the 1st 4 × 25-m (TP_2_), and after the 2nd maximal 4 × 25-m set (TP_3_). BLa and HR showed significant changes during all testing points in both protocols (*P* ≤ 0.01; *ES range*: 0.45–1.40). SmO_2_ was different only between TP_1_ and TP_3_ in both protocols (*P* ≤ 0.05–0.01; *ES* range: 0.36–1.20). A large correlation during the Max protocol between SmO_2_ and HR (*r*: 0.931; *P* ≤ 0.01), and also between SmO_2_ and BLa was obtained at TP_1_ (*r*: 0.722; *P* ≤ 0.05). A range of moderate-to-large correlations was revealed for SmO_2_/HR, and BLa/HR for TP_2_ and TP_3_ after both protocols (*r* range: 0.595–0.728; *P* ≤ 0.05) were executed. SmO_2_ is a novel parameter that can be used when aiming for a comprehensive evaluation of competitive swimmers' acute responses to sprint interval swimming, in conjunction with HR and BLa.

## Introduction

Monitoring training intensity is essential for evaluating athletes' response to an exercise program. A testing tool often utilized in sports environments as an intensity marker is blood lactate (BLa) concentration due to its sensitivity to detect training-induced changes (Beneke et al., 2011). Despite several potential limitations, including its invasive nature (Swart and Jennings, 2004), BLa testing has been extensively used in swimming to evaluate current performance status, and potentially predict future performance outcomes (Smith et al., 2002). Complementary to BLa testing, the percentage of maximum heart rate (HR) also makes an important contribution to assess training intensity (Borresen and Lambert, 2008), although characterized as not very informative regarding an athlete's training status (Buchheit, 2014). Moreover, the critical velocity may be used as a feasible and practical approach for monitoring swimming training intensity (Tijani et al., 2021).

Near-infrared spectroscopy (NIRS) is a relatively new technique with increasing popularity due to the fact that it non-invasively and directly enables measurements of changes in tissue oxygenation and hemodynamics as a response to dynamic exercise (Bhambhani, 2004). Recently, this technology has been applied in swimming as a complementary method to monitor peripheral training adaptations, to examine acute training responses to athletes of different competitive levels, and to evaluate different active recovery protocols (Jones et al., 2018; Dalamitros et al., 2019; Pratama and Yimlamai, 2020). In addition, NIRS has been examined as a potential alternative to BLa measurement in swimmers of different training levels (Wu et al., 2015). However, in this latter case, the testing procedure included an incremental dry-land test.

In swimming training, interval sets of various intensities are daily incorporated to activate either aerobic or anaerobic processes. As such, exploring the potential relationship of different testing procedures used to assess training intensity, namely, muscle oxygenation, HR, and BLa during submaximal and maximal efforts, could be important for both swim coaches and for sports scientists. Moreover, since it has been reported that warm-up protocols of different intensities induce different BLa but not HR responses on a subsequent maximal 100 m time-trial (Neiva et al., 2017), it would be interesting if such results were examined using muscle oxygenation testing. Thus, the purpose of this study was to evaluate and compare the interrelationship between muscle oxygenation (SmO_2_), HR, and BLa after a submaximal (Submax) or a maximal (Max) swimming interval protocol, and a main subsequent maximal interval set.

## Materials and Methods

### Subjects

A total of twelve national-level swimmers, nine male (*n* = 9; age: 21.9 ± 2.0 years; body mass: 78.8 ± 9.8 kg; body height: 182.7 ± 8.1 cm; FINA 2019 scoring points: 578.4 ± 89.0) and three female (*n* = 3; age: 20.2 ± 1.5 years; weight: 64.5 ± 6.7 kg; height: 174.3 ± 3.5 cm; FINA 2019 scoring points: 638.7 ± 23.0), from two different swimming clubs participated in this study. Swimmers were specialized in various race distances and swimming techniques. Fédération Internationale de Natation (FINA) scoring calculation was based on each athlete's specialty event according to short course's 2019 world records. Written informed consent was obtained from each participant. All procedures were in accordance with the Helsinki declaration and were approved by the Institutional Review Board.

### Methodology

Participants were engaged in two testing sessions. During the first session, anthropometric (body height and body mass) and training characteristics (distance specialty, preferred swimming technique, and best swimming times) were recorded. After completing a standardized in-water warm-up consisting of 1,200 m (continuous swimming/arm and kick drills/short sprints/cool down) following a 2 min passive rest, participants randomly performed either the Submax or the Max interval swimming protocol, in a counter-balanced order. Three days later, the second protocol was applied. Submax interval set consisted of 8 × 50 m intercepted with a 30 s passive rest, at an intensity corresponding to the critical velocity, which was calculated by 92% of the best performance during a maximal 400 m test (Zacca et al., 2016) conducted the week before the initiation of the study. During the Max interval protocol, swimmers performed a 4 × 15 m set starting at 1 min. Following both Submax and Max interval protocols, participants executed the main interval set consisting of 2 × 4 × 25 m at maximal intensity with a 30 s passive rest between each 25 m and 4 min between sets.

**Figure 1:**
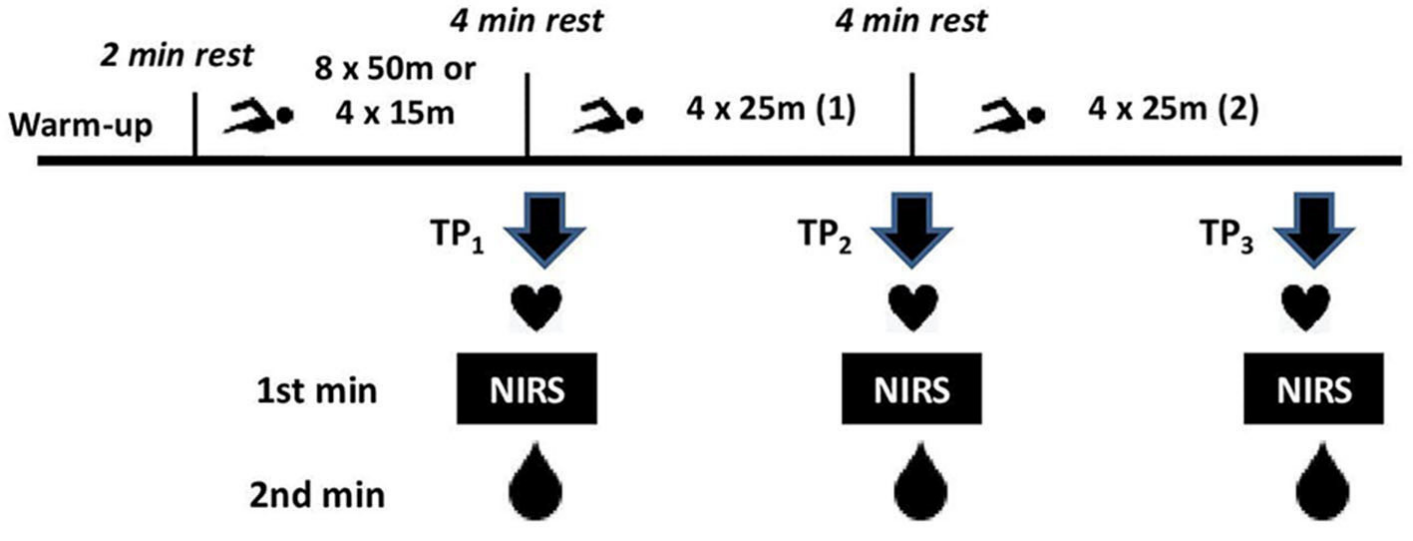
Schematic representations of the testing procedure. TP_1_ = testing point 1; TP_2_ = testing point 2; TP_3_ testing point 3.

Muscle oxygen saturation (SmO_2_) measurement was conducted with a portable near-infrared spectroscopy (NIRS) device (MOXY, Fortiori Design LLC, Hutchinson, Minnesota, USA). The SmO_2_ of the deltoid muscle of the dominant arm of each participant was measured in a sitting position, with the swimmer's arms hanging freely to the side and fully relaxed. The device was placed in the middle of the muscle belly, while the exact position was pointed with a permanent marker to place the monitor in the same spot for each measurement. All athletes presented skinfold thickness less than the accepted limit of 12 mm at the measurement point (Barstow, 2019). SmO_2_ of the relaxed muscle was recorded for 1 min at rest and the average values were analyzed. Subsequent recordings for SmO_2_ measurements took place during the 1st post-exercise minute, giving adequate time for athletes to exit the water at three specific testing points: following the Submax or Max protocols, (TP_1_), following the first 4 × 25 set, (TP_2_), and following the second 4 × 25 set (TP_3_). Simultaneously, during all tests, HR was recorded using chest belt telemetry (Polar S810 Electro, Kempele, Finland). To measure BLa, a portable analyzer (Lactate Scout 4, EKF Diagnostics, Germany) was used. BLa was collected at the second post-exercise min. SmO_2_ and BLa measurements were conducted by two experienced examiners under the same conditions. The testing procedure is summarized in Figure 1.

All swim tests were performed using a push-off start from within the water with the front-crawl technique. Swimmers were instructed to avoid underwater gliding. All procedures were conducted during the same training period (December) and in the daytime (8:00:00–9.30:00 h), under the same water temperature (26–27°C) in an indoor 25 m swimming pool. Swimmers were advised to follow the same training routine as well as diet, hydration, and sleeping habits the day before testing.

### Statistical Analysis

Kolmogorov-Smirnov test for normality, Pearson's correlation analysis, and analysis of variance (ANOVA) with repeated measures were conducted. SmO_2_, BLa, and HR data were analyzed using two-way ANOVA (*protocol*: Submax and Max × *time*: TP_1_, TP_2_, and TP_3_) with repeated measures on *time* factor. *Post-hoc* analyses were conducted using the Scheffé test. Correlation thresholds were classified as: <0.1 = *trivial*, <0.3 = *small*, <0.5 = *moderate*, <0.7 = *large*, <0.9 = *very large*, and ≤ 1.0 = *near perfect* (Hopkins et al., 2009). Effect size (*ES*) values of ≤ 0.2, between 0.21, and 0.8, and >0.8 were considered as small, moderate, and large, respectively (Cohen, 1988). The statistical significance level was set at *p* ≤ 0.05. All statistical analyses were conducted using SPSS 25.0 software (IBM, NY, USA). Data are presented as mean ± standard deviation (*SD)*.

## Results

No effect of *protocol* was found (*p* = 0.198) in any of the measured parameters. In contrast, a significant main effect of *time* was revealed (*p* < 0.001). HR and BLa were increased between all three testing points at both protocols (*p* < 0.05; *p* < 0.001, *ES* range: 0.36–1.40). SmO_2_ values were only different between TP_1_ and TP_3_ (*p* < 0.05 and 0.001; *ES*: 1.09 and 1.20, for the Submax and Max protocols, respectively), but not either TP_1_ and TP_2_ or TP_2_ and TP3 after both protocols (*p* > 0.05) (Table 1).

**Table 1:** Statistical significance, effect size, muscle oxygen saturation, heart rate, and blood lactate values at all testing points during both protocols.

**Protocol**	**Variables**	**TP_**1**_**	**TP_**2**_**	**TP_**3**_**	***P***	***ES***
Submax	SmO_2_ (%)	59.4 ± 9.1	52.2 ± 10.6	48.3 ± 11.2	0.046^a^	0.36–1.09
	HR (b·min^−1^)	154 ± 19.7	170 ± 8.8	174 ± 8.9	0.003^a^^,^^b^	0.45–1.40
	BLa (mmol·L^−1^)	3.5 ± 1.4	8.4 ± 2.5	11.8 ± 3.0	0.000^c^^,^^d^^,^^e^	0.52–1.24
Max	SmO_2_ (%)	57.0 ± 6.4	47.8 ± 9.0	37.8 ± 11.4	0.001^c^	1.19–1.20
	HR (b·min^−1^)	145 ± 14.2	167 ± 11.0	172 ± 6.4	0.000^c^^,^^d^^,^^e^	0.57–0.22
	BLa (mmol·L^−1^)	4.4 ± 1.5	9.5 ± 2.6	12.7 ± 2.9	0.000^c^^,^^d^^,^^e^	0.49–1.16

*Submax protocol: 8 × 50 m; Max protocol: 4 × 15 m; TP_1_, Testing point after the 8 × 50 m or the 4 x 15 m; TP_2_, Testing point after the 1st 4 × 25 m; TP_3_, Testing point after the 2nd 4 × 25 m; SmO_2_, muscle oxygenation; BLa, blood lactate concentration; HR, heart rate;*

a *TP_1_ significantly different from TP_3_ (p < 0.05)*,

b *TP_1_ significantly different from TP_2_ (p < 0.05);*

c *TP_1_ significantly different from TP3 (p < 0.001);*

d *TP_1_ significantly different from TP_2_ (p < 0.001);*

e *TP_2_ significantly different from TP_3_ (p < 0.001)*.

Muscle oxygen saturation (SmO_2_) and BLa values were *highly* correlated at TP_1_ during the Max protocol (*r* = 0.722; *p* < 0.05), while *moderate* correlations were found at TP_2_ and TP_3_ (*r* = 0.488 and 0.498; *p* > 0.05). HR and SmO_2_ showed a range of *moderate*-to-*high* correlation magnitudes during the three testing points at both protocols (*r* range: 0.645–0.728; *p* < 0.01), while a *very high* correlation was obtained at TP_1_ after the Max protocol (*r* = 0.931; *p* < 0.01). Similarly, BLa and HR correlation coefficient were also *moderate*-to-*high* at all testing points in both protocols (*r* range: 0.595–0.694; *p* < 0.05). Finally, *small* correlations were observed between SmO_2_ and BLa during the Submax protocol at all testing points (*r* range: 0.147–0.285; *p* > 0.05) (Table 2).

**Table 2:** Pearson's correlation magnitudes between the different testing procedures at all testing points during both protocols.

**Submax protocol**								
**TP**_**1**_			**TP**_**2**_			**TP**_**3**_		
	**BLa**	**HR**		**BLa**	**HR**		**BLa**	**HR**
SmO_2_	0.147	0.683^*^	SmO_2_	0.202	0.695^*^	SmO_2_	0.285	0.645^*^
BLa		0.595^*^	BLa		0.660^*^	BLa		0.679^*^
**Max protocol**								
**TP**_**1**_			**TP**_**2**_			**TP**_**3**_		
	BLa	**HR**		**BLa**	**HR**		**BLa**	**HR**
SmO_2_	0.722^*^	0.931^**^	SmO_2_	0.488	0.723^*^	SmO_2_	0.498	0.728^*^
BLa		0.694^*^	BLa		0.694^*^	BLa		0.622^*^

*Submax protocol: 8 × 50 m; Max protocol: 4 × 15 m; TP_1_: Testing point after the 8 × 50 m or the 4 x 15 m; TP_2_, Testing point after the 1st 4 × 25 m; TP_3_, Testing point after the 2nd 4 × 25 m; SmO_2_, muscle oxygenation; BLa, blood lactate concentration; HR, heart rate*.

* *p < 0.05;*

** *p < 0.01*.

## Discussion

The application of portable near-infrared spectroscopy technology in the sport performance area is progressively increasing. The present study demonstrated that the muscle oxygenation variable evaluated (SmO_2_) was mainly correlated with BLa and HR values after the Max protocol. That is, immediately after the completion of a very low volume sprint interval set (4 × 15 m, duration of 7–8 s).

A significant correlation between SmO_2_ and BLa values has been previously described in swimmers during incremental testing performed on dry land. In this case, the application of NIRS technology was suggested as a non-invasive alternative to BLa testing (Wu et al., 2015). The novelty of our study is that, for the first time, this interrelationship was examined during interval efforts based on anaerobic and aerobic metabolism that are regularly applied in swimming training.

Understanding muscle physiology during dynamic exercise is essential for evaluating exercise intensity. SmO_2_ values of the deltoid muscle during front-crawl swimming provided a clear representation of the balance between O_2_ delivery and extraction of the body's part which mostly functions during horizontal propulsion (Morouço et al., 2015). BLa, on its part, is sensitive to changes in exercise intensity and duration (Beneke et al., 2011). On the other hand, real-time data accumulation through NIRS is a useful evaluation tool during training efforts (Jones et al., 2018). Thus, the conjunction of the two testing procedures may prove beneficial for accurately and thoroughly evaluating intensity during swimming. In the present study, muscle oxygenation was reduced progressively regardless of the intensity of the “priming” exercise (Submax or Max protocols). However, a limitation of the present study may be recognized by the post-swimming NIRS measurement. This was applied to avoid any movement of the apparatus on the muscle during fast arm movements. One-minute post-swim values are expected to be higher compared to the values during swimming. In this case, swimming and recovery rate values may be different between protocols, but this was not detected with a single recovery sampling, thus affecting the correlation between SmO_2_ with BLa and HR. On the other hand, collecting recovery values makes the measurement more practical and feasible to use during training.

Swimming coaches and sports scientists usually apply field tests during both training and competition. In this sense, BLa and HR measurements serve as “standard” physiological testing procedures. Acknowledging that different responses, especially in high training efforts, have been shown during BLa and HR testing in swimming (Skorski et al., 2012), their usage in combination with near-infrared spectroscopy measurement can be realized as a form of alternative or complementary method, depending on the performed exercise intensity. Moreover, it may potentially offer a non-invasive analysis of dynamic changes in oxygenation and blood volume, detect the relative muscles contribution, and assess training-induced adaptations after endurance training (Jones and Cooper, 2016; Jones et al., 2018). Future studies should consider this relationship in swimming distances of longer duration.

The interpretation and practical translation of the data collected from the NIRS portable device is probably the biggest challenge when this type of technology is applied. Information on skeletal muscle oxygen levels can increase the understanding regarding the internal load of both active and less active muscles as evident in the case of two or more monitors being involved during training and recovery periods (Manchado-Gobatto et al., 2020). Moreover, high muscle deoxygenation values, like those obtained during sprint interval sets, may be linked to greater peripheral adaptations (Paquette et al., 2019) or may even characterize the training status among individuals (Ding et al., 2001). Overall, NIRS method is presented as an appropriate solution for quick and continuous field-based evaluation in a variety of sports, thus, assessing both acute and chronic adaptations, while characterized by high sensitivity in different exercise demands and good reproducibility values (Perrey and Ferrari, 2018). Still, protocol standardization is vital considering the existing limitations, such as the impact of adipose tissue thickness and the need for suitable physiological calibration (McManus et al., 2018; Barstow, 2019).

The application of the NIRS technology to monitor muscle oxygenation responses in this study (MOXY monitor) has been recently used in different sport activities, including sprint kayaking, sport climbing, and cross-country skiing. In general, these studies highlighted the potential of this research tool to provide information regarding peripheral adaptations following high-intensity interval training (Paquette et al., 2019, 2021), SmO_2_ availability in different exercise intensities (Feldmann et al., 2020), and muscle activation of upper and lower muscle groups during a long distance race (Stöggl and Born, 2021). In this study, the implementation of a low volume maximal intensity set (2 × 4 × 25 m) was driven by previous findings indicating significant BLa increases with a similar training stimulus (Kabasakalis et al., 2020), while the rest of the intervals were guided by the need to perform the measurements. The specific Submax and Max protocols applied were chosen based on stimulating different metabolic energy systems. In accordance with a previous swimming study that analyzed the responses of different warm-up intensities on BLa and HR levels (Neiva et al., 2017), both Submax and Max protocols concluded no significant variations on the respective values in any of the three testing points. Therefore, it can be suggested that physiological testing during maximal short interval performance is not affected by previous “pre-activation” protocols.

In conclusion, after maximal swimming protocols consisted of very short (i.e., 15 m) and short interval efforts (i.e., 25 m), a high interrelationship between values of muscle oxygenation as expressed by muscle oxygen saturation, heart rate, and blood lactate testing were revealed as compared to those obtained after an identical protocol where lower intensity interval efforts were initially applied.

## Data Availability Statement

The original contributions presented in the study are included in the article/supplementary material, further inquiries can be directed to the corresponding author/s.

## Ethics Statement

The studies involving human participants were reviewed and approved by Vasilis Mougios, Aristotle University of Thessaloniki, Department of Physical Education & Sport Science, Evangelia Kouidi, Aristotle University of Thessaloniki, Department of Physical Education & Sport Science Giorgos Grouios, Aristotle University of Thessaloniki, Department of Physical Education & Sport Science. The patients/participants provided their written informed consent to participate in this study.

## Author Contributions

AD, ES, and AK collected the data. ES and AK analyzed the data. AD, ES, and AK wrote the manuscript. AT revised the manuscript. All authors listed have made a substantial, direct, and intellectual contribution to the work and approved it for publication.

## Conflict of Interest

The authors declare that the research was conducted in the absence of any commercial or financial relationships that could be construed as a potential conflict of interest.

## Publisher's Note

All claims expressed in this article are solely those of the authors and do not necessarily represent those of their affiliated organizations, or those of the publisher, the editors and the reviewers. Any product that may be evaluated in this article, or claim that may be made by its manufacturer, is not guaranteed or endorsed by the publisher.
